# Progress in Diabetes

**Published:** 1903-12-26

**Authors:** 


*(To be continued.)*

### PROGRESS IN DIABETES.

In an interesting discussion of the pathology by Pflüger in his *Archives* for the present year[1] it is suggested that the change of glycogen into sugar is stimulated by impulses coming from various parts of the body as their need for sugar rises or falls, and on the other hand it is checked by the antidiastase formed in the pancreas. On the balance of these forces the absence of diabetes depends. The local stimuli usually act through the splanchnic nerves, but in some cases by secretions poured into the blood. He adheres to the belief that the transformation is effected by a ferment, and seems to overlook the strong evidence which has been brought against the presence of such a ferment. Rumpf[2] shows that the theory that there is a constant ratio of 2·8 to 1 between the sugar and urea excreted in diabetes is untrue, and in one case found that the ratio was as high as 6·8 to 1. Hesse, too, was able to get a high ratio by giving a diet in which fat predominated over the albuminoids. One suggestion of Rumpf's is important, that coma may be due to the loss of water and dehydration of the tissues. A. R. Elliott,[3] in discussing albuminuria, maintains that it is due either to heart failure or to toxic or degenerative changes. Now the toxic form is usually acute and precedes coma. It is caused by hyaline changes in the tubular epithelium the result of acid toxins. The degenerative form is due to a chronic nephritis in mild types of diabetes which is only of importance after it has continued for long periods. The question of the connection between oxaluria and diabetes is still undecided. J. Scott[4] carried out a series of experiments by injecting small doses of potassium oxalate, which was found to increase the nitrogenous metabolism. No sugar was found in the urine at any time, though tests for a longer period are needed. D'Estrée of Contrèxeville[5] insists on the common origin of gout and diabetes of a certain type, and points out that the excretion of uric acid in the form of crystals or gravel which follows a course of Contrèxeville water is accompanied in those patients with a fall or disappearance of the sugar. Beddard and others[6] show that in coma the alkalinity of the blood and the amount of $CO_2$ present are diminished. A rigid diet may produce a similar change, which may be removed by giving alkalies and a carbohydrate diet. Mosse[6] urges the utility of potatoes in the diet of diabetics. Thus in place of 6 oz. of wheaten bread, 15 to 18 oz. of baked potatoes, weighed raw, may be given. Even large amounts of potatoes may be taken by some patients. The sugar in his cases always decreased when potatoes were given, but did not vanish. Thirst and neuralgias decreased and the strength improved. Whether this starch is more easily stored than that from wheat or whether the potato contains any remedial substance is not clear. In cases of gangrene Lilienthal[8] recommends that if there is acid toxæmia threatening, no operation should be done, except to lay open any abscesses and to drain the tissues. If, however, there is no acetone or diacetic acid in the urine, and the use of an entire limb is threatened, he would operate above the upper level of the gangrene at a point where the best muscular flaps can be obtained. If a finger or toe is destroyed it should be simply removed, the phlegmon laid open, and the tendons and fasciæ allowed to slough away under wet dressings.

S. Hart and W. J. Gies[9] give observations on the amount of sugar, $\beta$-oxybutyric and diacetic acid (as shown by the quantity of ammonia) excreted by a patient during one month. These acids together with acetone were present throughout. Sugar increased when aspirin was given. The nitrogen due to ammonia when the diet was restricted reached four times the normal, and even 25 per cent. of the total nitrogen. This over-production is regarded as an effort to neutralise the excess of organic acids present. It was noted that when bicarbonate of soda was given to ward off the threatening coma the amount of ammonia diminished of itself. A. E. Finney[10] gives a valuable summary of the work that has been done with regard to the islands of Langerhans, more especially in connection with diabetes. These structures were first described in 1869, and consist of small spherical masses of epithelial cells, just visible to the naked eye, lying in irregular columns separated by tortuous vessels, and are most numerous in the tail and next in the body of the pancreas, where they are distributed among the acini, though they have no connection with excretory ducts. Laguesse in 1893 first suggested that they furnish the secretion which regulates the metabolism of the carbohydrates. In the embryo they are formed even before the acini, and remain more numerous in childhood than in adult life. Now as long as a portion of the pancreas remains in the body, pancreatic diabetes does not occur, and even ligature of the duct fails to produce it. The explanation seems to be that since after ligature the islands remain intact, though the rest of the gland wastes away, the islands supply the needful secretion. Though many cases of diabetes have been found in which the islands were normal, yet in a large proportion marked changes were present. The author's dissections of 35 cases lead him to the conclusion that morbid changes in the islands may take place independently of those in the rest of the pancreatic tissue, at least to a large extent; that diabetes may occur when the islands alone are affected by morbid changes; but on the other hand the disease may be found when the islands are apparently healthy, and in this case the affection may be of nervous origin or of the alimentary type. Slight lesions may be found where no diabetes exists if a sufficient number of healthy islands remain to carry on the work. The discussion of the functions of these islands of Langerhans has been joined in by numerous writers, especially during the last three years, while its importance has been increased by the discovery of adrenalian glycosuria. Herter, Blum, and others have shown that over-stimulation of the adrenals or injection of their secretion produces a remarkable excretion of sugar in the urine, while the most minute quantity painted on the pancreas will suffice to bring about its appearance. The question arises, Do the adrenals inhibit, or are they in any way capable, of neutralising the pancreatic functions? Besides this issue, there are the important questions raised by A. C. Croftan, who endeavours to prove that the internal pancreatic secretion is absorbed by the leucocytes and conveyed by them to all parts of the body. This secretion has the power of breaking up the molecule of sugar, but only in the presence of hæmoglobin, while the latter substance yields bile pigments and other bodies. Hence the rare instances of bronzed diabetes, of which some 27 are on record, and the allied cases of hæmochromatosis which Von Recklinghausen, Opie, and others have recently discussed at length where there is general deposit of iron pigment without diabetes.

1 Scottish Med. and Surg. Jour., Sept. 2 Birmingham Med. Rev, July. 3 Lancet, Sept. 11. 4 Brit. Med. Jour., July 4. 5 Lancet, May 9. 6 Ibid, July 11. 7 Dublin Jour. Med. Sci., July. 8 New York Med. Jour., July 4. 9 Amer. Jour. Med. Sci., p. 305. 10 Med. Chron., Vol. II., p. 137.

*(To be continued.)*

